# Supplementary material for: Living Chain-Walking (Co)Polymerization of Propylene and 1-Decene by Nickel α-Diimine Catalysts
Source: Polymers (Basel). 2020 Aug 31;12(9):1988. doi: 10.3390/polym12091988 (PMC7564000; doi:10.3390/polym12091988)
Supplement: Supplementary file 1 [file polymers-12-01988-s001.pdf]

Supplementary Materials: Living Chain-Walking (Co)Polymerization of Propylene and 1-Decene by Nickel α-Diimine Catalysts

Pei Li, Xiaotian Li, Shabnam Behzadi, Mengli Xu, Fan Yu, Guoyong Xu and Fuzhou Wang

1. Procedure for the synthesis of ligands L1–L4 and complexes 1–4

Synthesis of *para*-phenyl substituted *α*-diimine ligands **L1–L4** and their nickel complexes **1–4** are outlined in Scheme S1. The Suzuki coupling reaction of the bromoaniline derivatives and phenylboronic acid obtained with a Pd(II) catalyst led to the corresponding phenyl-substituted aniline derivatives **1′–4′**.[S1,S2] The ligands **L1** and **L2** were prepared by the condensation of equivalents of the appropriate aniline with one equivalent of acenaphthoquinone, usually in the presence of a formic acid as a catalyst. The desired ligands **L3** and **L4** were prepared by the the Schiff base condensation using *p*-toluenesulfonic acid (*p*-TsOH) as catalyst in high yields (Scheme S1). All *α*-diimine ligands **L1–L4** [S1,S3] were characterized by elemental analysis, ^{1}H and ^{13}C NMR. Complexes **1–4** were synthesized from the reactions of the corresponding ligand with (DME)$NiBr_2$ in high yields. These complexes were characterized by IR spectroscopy and elemental analysis.

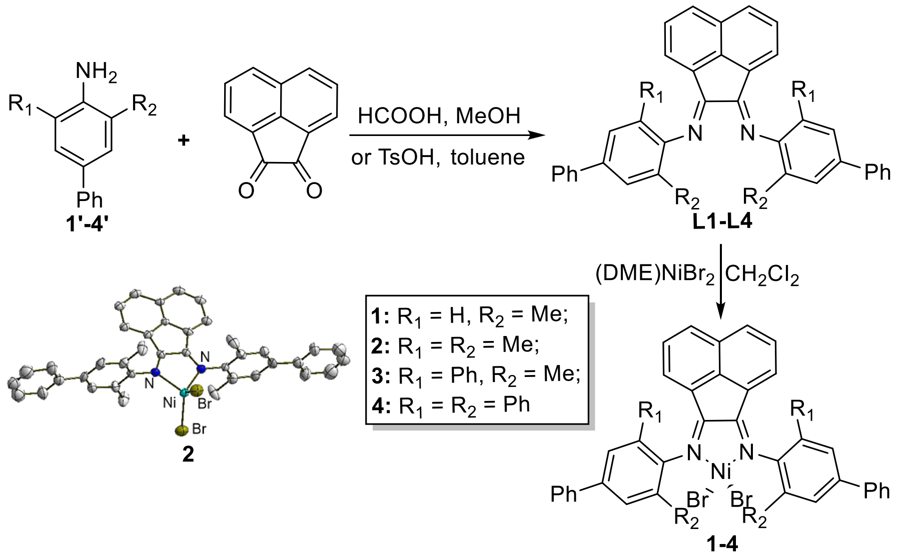

Scheme S1. Synthesis of *para*-phenyl substituted *α*-diimine ligands **L1–L4** and their complexes **1–4**.

2. X-ray crystallography for complex 3

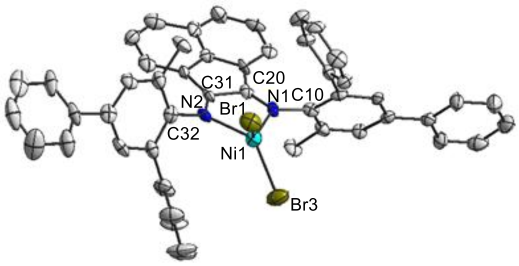

Table S1. Crystal data and structure refinement for complex **3**.

Complex	**3**
CCDC	1851985
Empirical Formula	$C_{50}H_{35}Br_2N_2Ni$
Formula mass	882.33
Temperature (*K*)	296
Wavelength (*Å*)	0.71073
Crystal size (*mm*^{3})	0.29 × 0.27 × 0.25
Crystal system	Monoclinic
Space group	$P2_1/c$
a (Å)	11.171 (9)
b (Å)	21.708 (18)
c (Å)	18.085 (15)
V (Å^3)	4367 (6)
Z	4
Density (calcd.) (mg/cm^3)	1.342
Absorption coefficient (mm^{-1})	2.31
F(000)	1788
Theta range for data collec. (°)	2.3–18.1
Limiting indices	$-12 \le h \le 13$
	$-26 \le k \le 25$
	$-21 \le l \le 17$
No. of rflns collected	7760
No. unique rflns [R(int)]	2710
R_{int}	0.115
Final *R* indices [$I > 2\sigma(I)$]	$R_1 = 0.0804$
	$wR_2 = 0.1376$
R indices (all data)	$R_1 = 0.2013$
	$wR_2 = 0.1558$
Goodness-of-fit on F^2	1.018
Max. and min. transmission	0.5541 and 0.5961
Largest diff. peak and hole (e.Å^{-3})	0.648 and –0.602

3. Equations for the microstructure analysis of the (co)polymers

Calculation of the degree of branching

The degree of branching (B) was estimated by ^{1}H NMR spectroscopy[S1,S4] and was corrected for end groups as follows:

$$B = \frac{2(I_{CH_3})}{3(I_{CH} + I_{CH_2} + I_{CH_3})} \times 1000 \qquad \text{Eq. S1}$$

Branching degree, the number of methyl carbon in every 1000 carbons, CH_3, CH_2, CH refer to the intensities of the methyl, methylene and methine resonances in ^{1}H NMR spectra.

Calculation of the total CH_3 and each branch

The ^{13}C NMR spectra of the (co)polymers showed the resonances of methyl, ethyl, propyl, butyl and longer than amyl branches. By the use of these resonances of the branching carbans, i.e., $1B_1$, $1B_2$, $1B_3$, $1B_4$, $2B_3$ and $2B_4$, the contents of the total CH_3 and each branch can be determined by the following equation:[S1,S4]

$$\mathbf{CH_3} = I_{1B_1} + I_{1B_2} + \left(I_{1B_3} + I_{3B_3}\right)/2 + I_{1B_4} + I_{Lg}$$

Methyl branch, $\mathbf{Me} = I_{1B_1} + I_{2B_3} - I_{1B_3}$; Ethyl branch, $\mathbf{Et} = \left(I_{1B_2} + I_{2B_2}\right)/2$

Propyl branch, $\mathbf{Pr} = \left(I_{1B_3} + I_{3B_3}\right)/2$; Butyl branch, $\mathbf{Bu} = \left(I_{1B_4} + I_{2B_4}\right)/2$ Eq. S2

Longer than amyl branches, $\mathrm{Lg} = \left(I_{1B_n} + I_{2B_n} + I_{3B_n}\right)/3$

Where branching numbers per 1000 carbon atoms were determined by ^{13}C NMR spectroscopy.

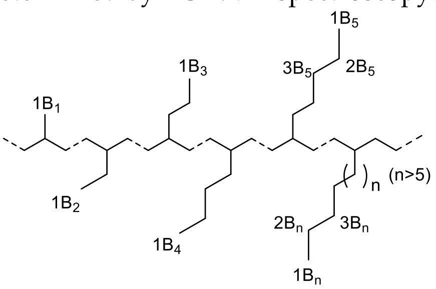

Calculation of ω,1-insertions

The fraction of ω,1-insertions in of the poly(1-alkene) was calculated using the following equation reported by Brookhart *et al*.[S5]

$$\omega{,}1\% = \frac{1000 - (\omega - 2)B}{1000 + 2B} \times 100 \qquad \text{Eq. S3}$$

Where B is the total branching calculated by **Eq. S1**, ω is the number of carbon atoms in the monomer.

4. ¹H and ¹³C NMR spectra of the obtained polypropylenes (Table 1)

PP:

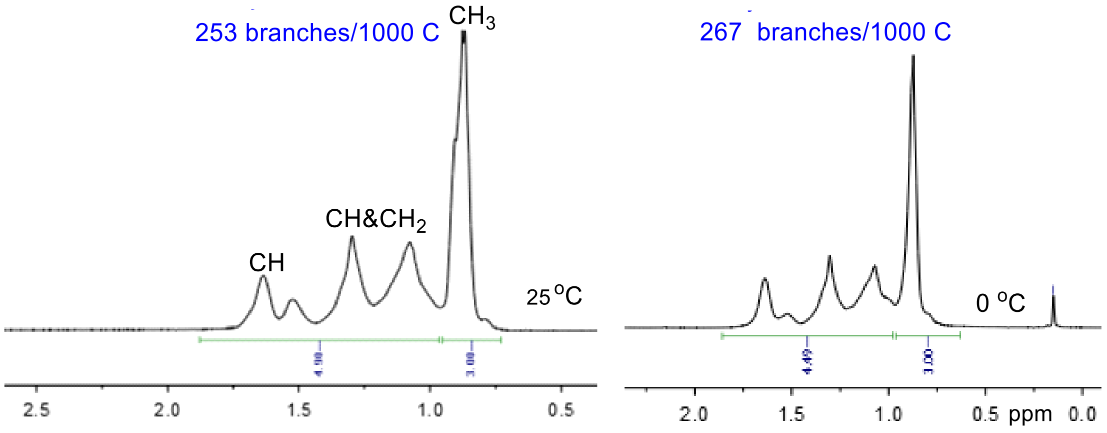

Fig. S1. ¹H NMR spectra of the polypropylenes obtained with **2**-MMAO at 0 and 25 °C (entries 1 and 2, Table 1).

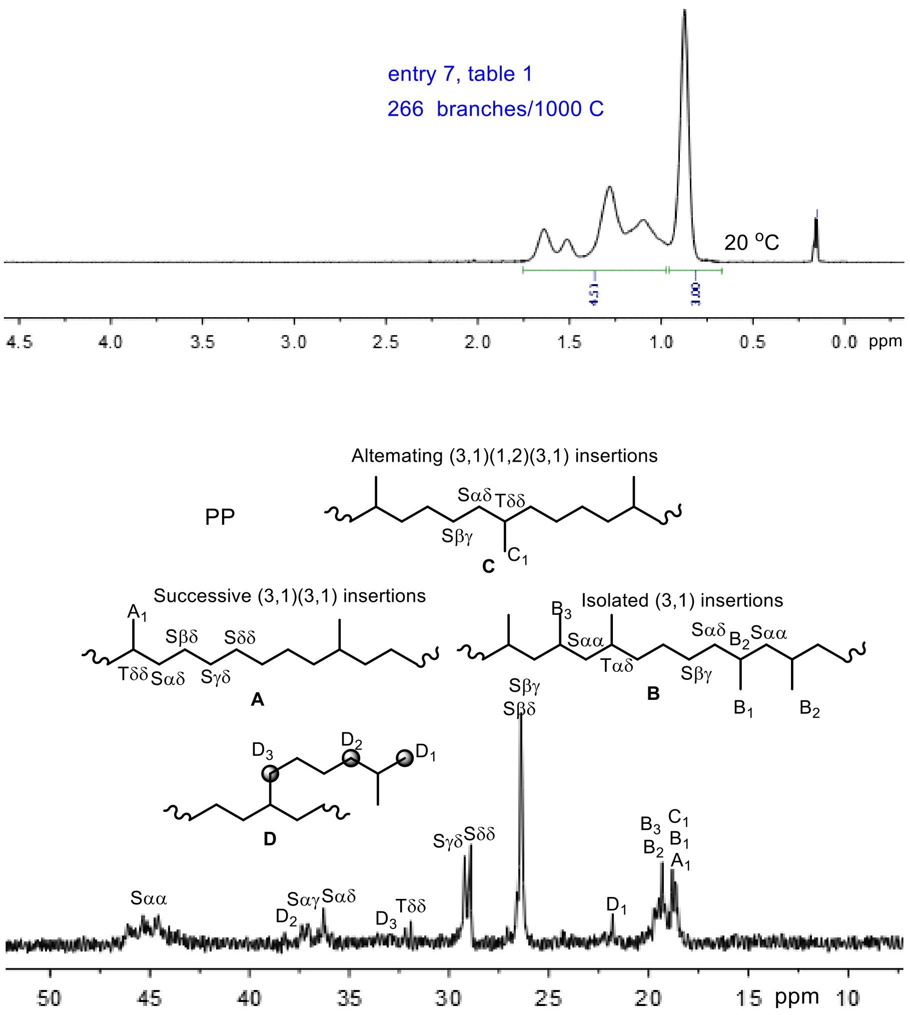

Fig. S2. ¹H and ¹³C NMR spectra of the polypropylene obtained by **5**-MMAO at 25 °C (entry 7, Table 1).

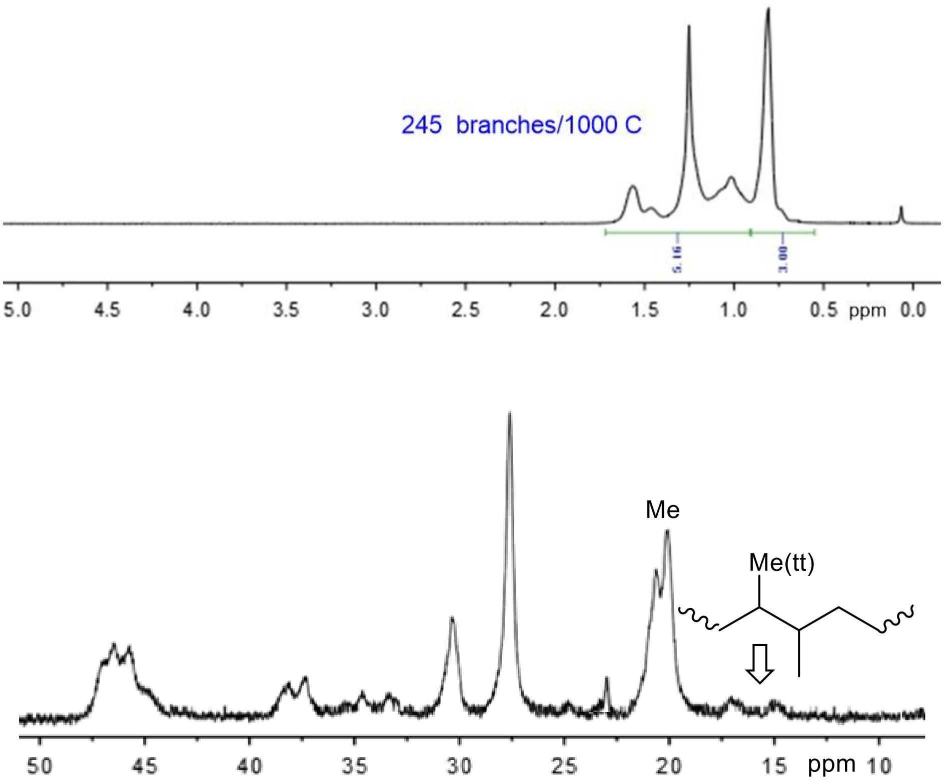

Fig. S3. ^{1}H and ^{13}C NMR spectra of the polypropylene obtained by **2**-MMAO at 25 °C for 50 min (entry 11, Table 1).

5. ^{1}H and ^{13}C NMR spectra of the obtained P/1-decene copolymers (Table 3**)**

Poly(P-*co*-D):

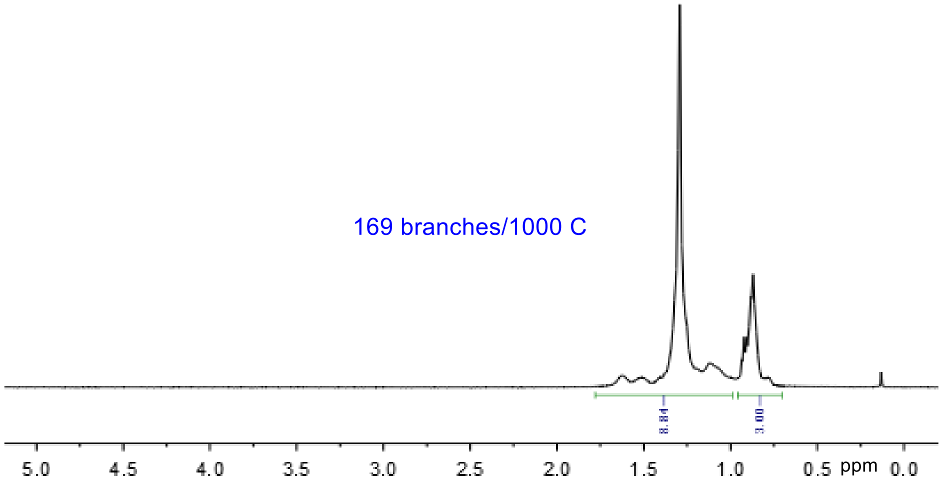

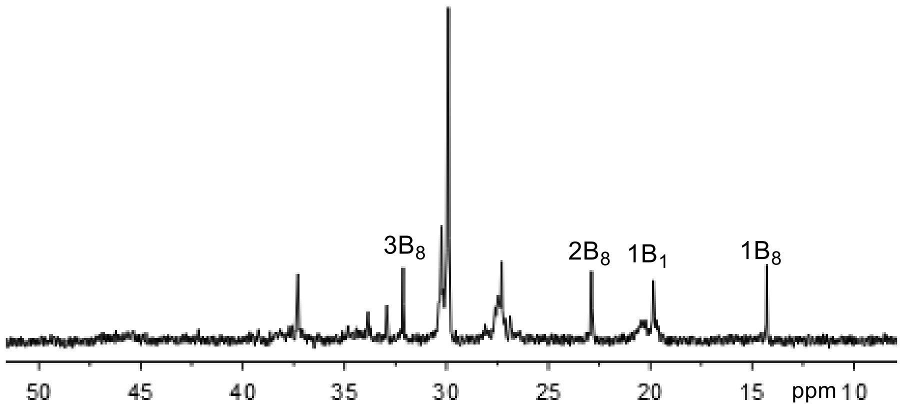

Fig. S4. ^{1}H and ^{13}C NMR spectra of the poly(P-*co*-D) obtained by **2**-MMAO at 25 °C (entry 1, Table 3).

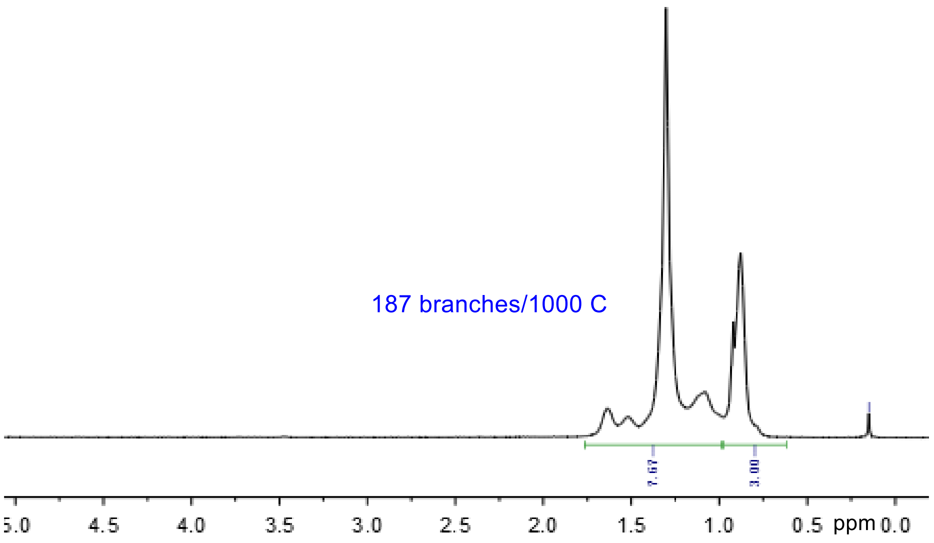

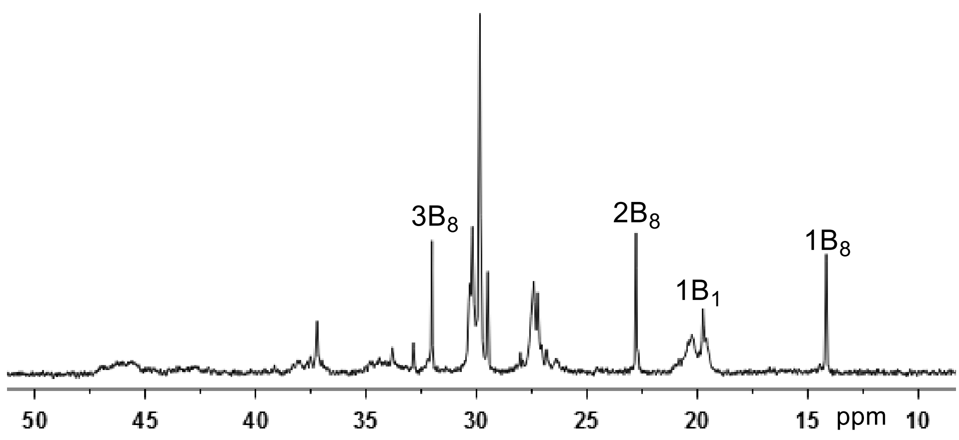

Fig. S5. ^{1}H and ^{13}C NMR spectra of the poly(P-*co*-D) obtained by **2**-MMAO at 0 °C (entry 2, Table 3).

6. GPC curves of the polypropylenes

Living polymerization of propylene with **2**-MMAO at 25 °C (Fig. 2 (i)):

5 min

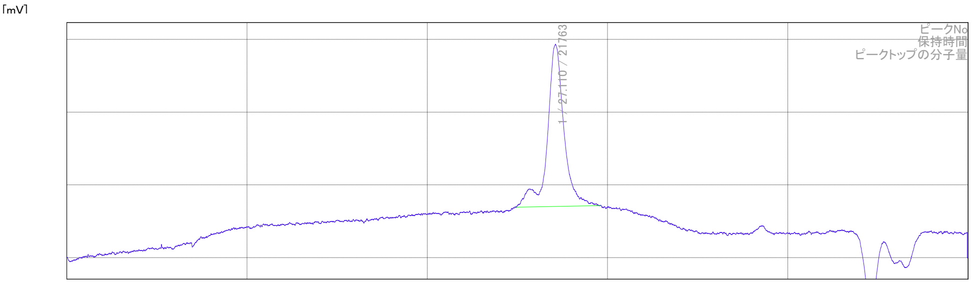

RSLT0002 : 2014-12-25.chd : wfz-2-5
: クロマト RI (-0.588 - 6.460 [mV])

- RI -	Mn:	20832	Mw:	23310	Mz:	26671	Mw/Mn:	1.119

10 min

entry 8,

Table 1

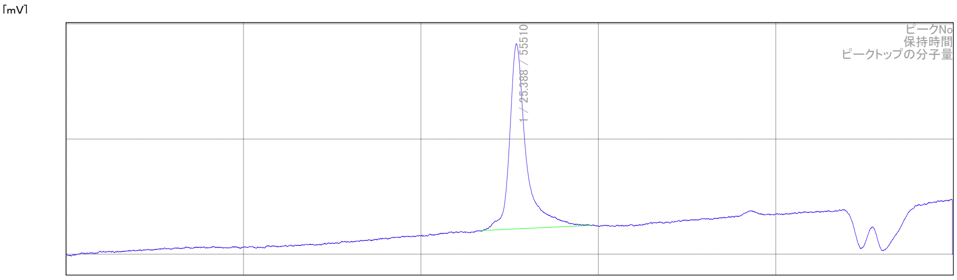

RSLT0004 : 2014-12-22.chd : wfz-pp-no-80
: クロマト RI (-0.903 - 10.055 [mV])

- RI -	Mn:	43973	Mw:	52380	Mz:	58924	Mw/Mn:	1.191

20 min

entry 9,

Table 1

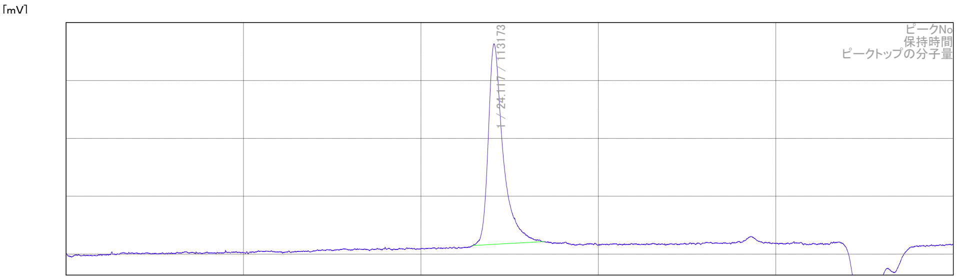

RSLT0002 : 2014-10-02.chd : wfz-no-34
: クロマト RI (-0.719 - 8.000 [mV])

- RI -	Mn:	94374	Mw:	104067	Mz:	111897	Mw/Mn:	1.103

30 min

entry 2,

Table 1

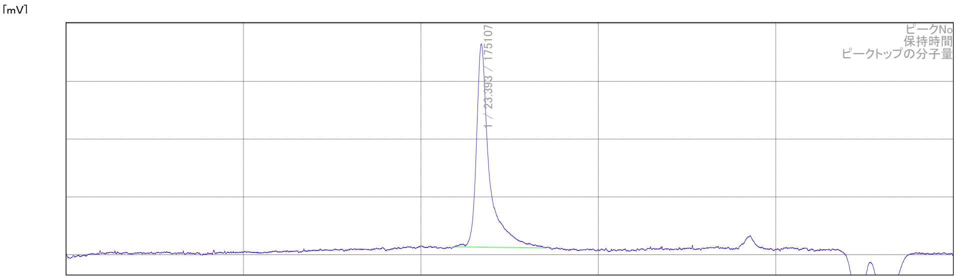

RSLT0004 : 2014-12-25.chd : wfz-pp-15
: クロマト RI (-0.701 - 8.032 [mV])

- RI -	Mn:	134102	Mw:	156026	Mz:	170396	Mw/Mn:	1.163

40 min

entry 10,

Table 1

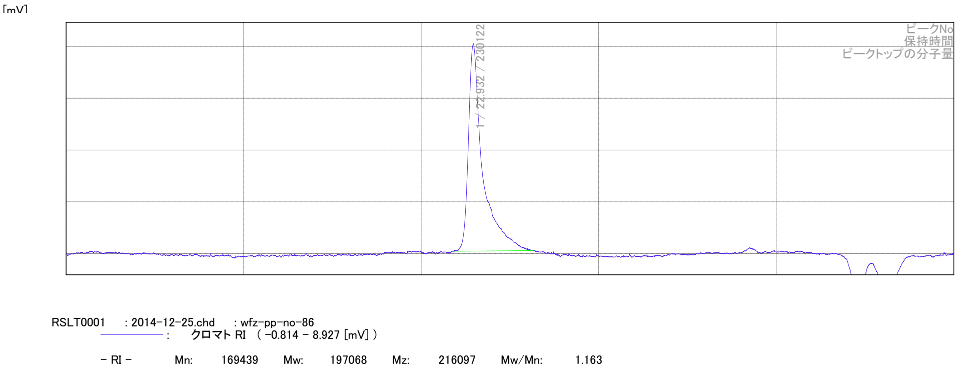

50 min

entry 11,

Table 1

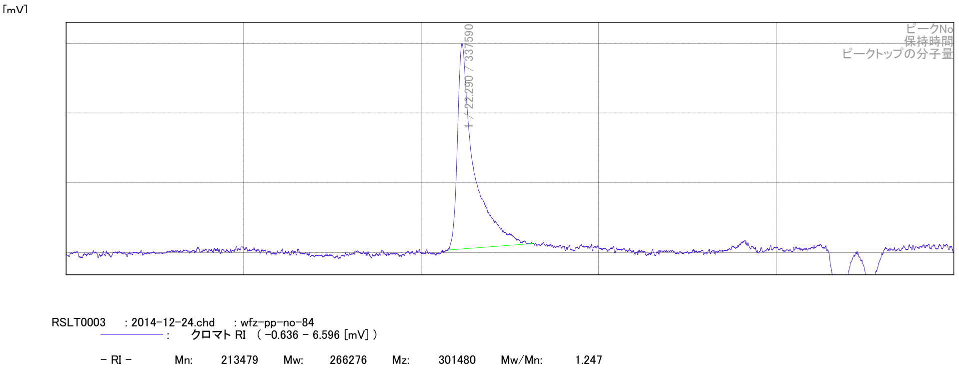

Fig. S6. GPC traces for the PPs obtained by **2**-MMAO at 25 ^{o}C for 5–50 min (Fig. 2 (i), entries 2 and 8–11, Table 1).

0 ^{o}C entry

1, Table 1

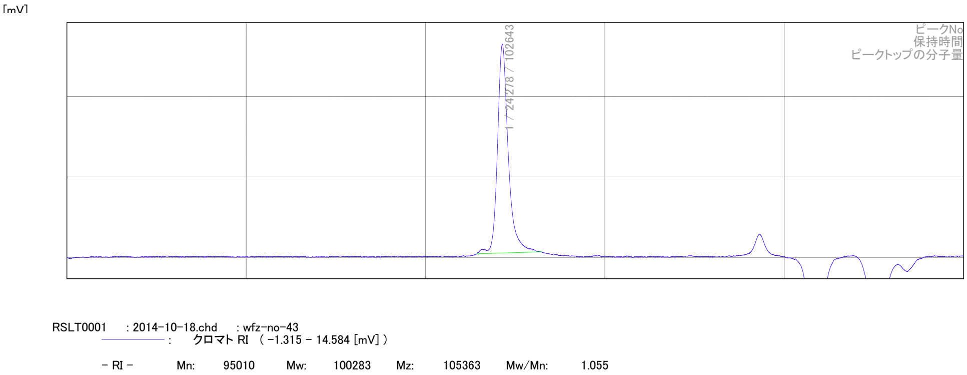

Fig. S7. GPC trace for the PP obtained by **2**-MMAO at 0 ^{o}C for 30 min (entry 1, Table 1).

7. GPC curves of the P/1-decene copolymers

Living copolymerization of propylene and 1-decene with **2**-MMAO at 25 ºC (Table 4), GPC traces for the poly(P-*co*-1-decene)s (Fig. 2 (ii)):

5 min

entry 1,

Table 4

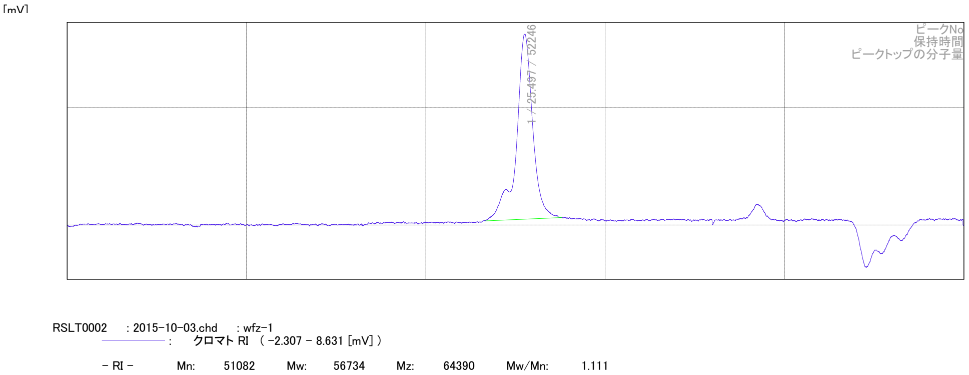

10 min

entry 2,

Table 4

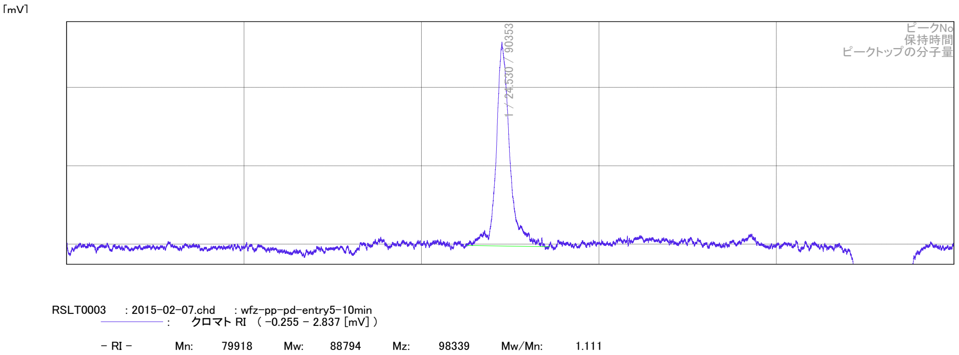

30 min

entry 3,

Table 4

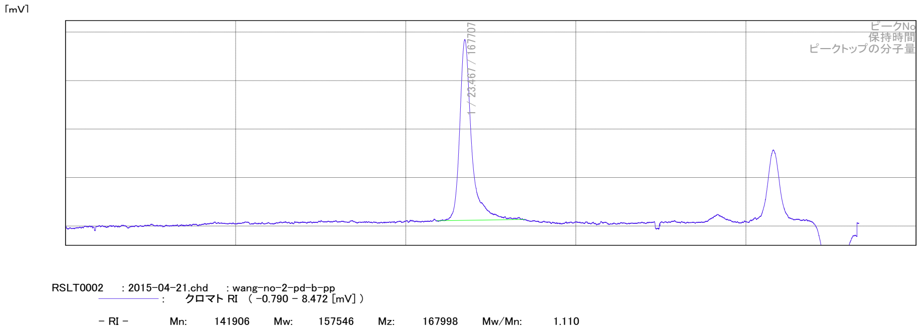

50 min

entry 4,

Table 4

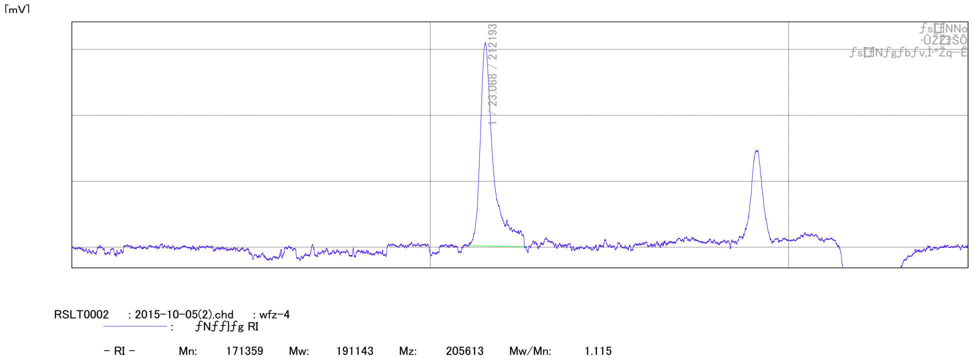

70 min

entry 5,

Table 4

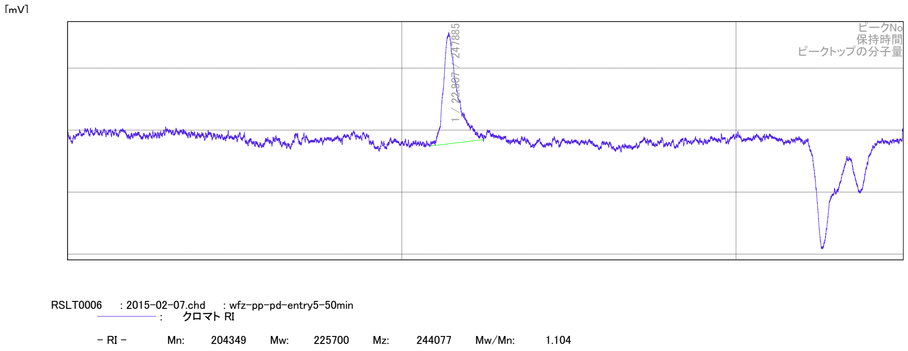

90 min

entry 6,

Table 4

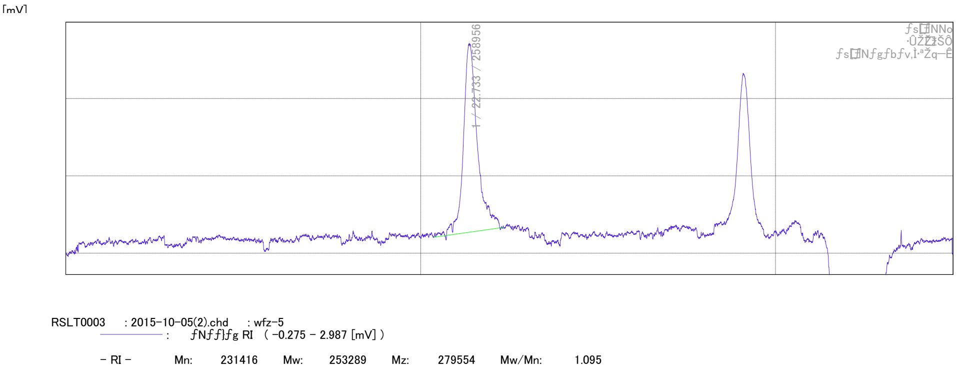

Fig. S8. GPC traces for the poly(P-*co*-D)s obtained by **2**-MMAO at 25 °C for 5–90 min (Fig. 2 (ii), entries 1–6, Table 4).

Poly(P-*co*-D):

25 °C

entry 1,

Table 3

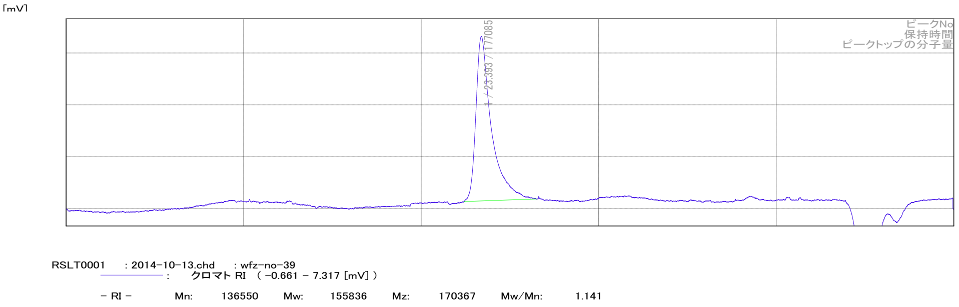

0 °C

entry 2,

Table 3

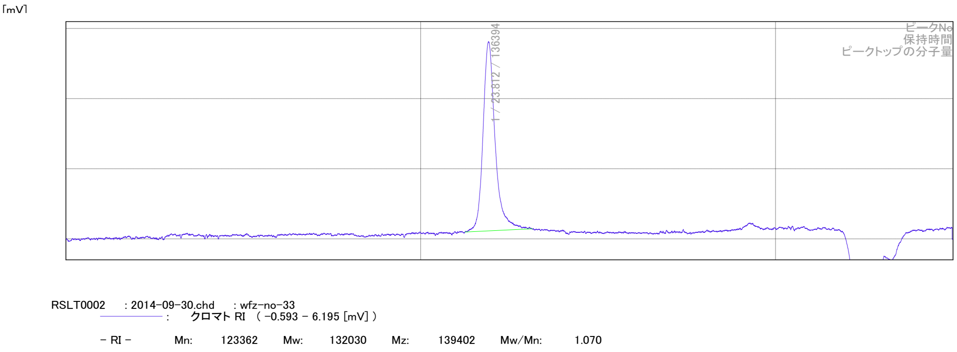

Fig. S9. GPC traces for the poly(P-*co*-D)s obtained by **2**-MMAO at 0 and 25 °C (entries 1 and 2, Table 3).

8. DSC curves of the polypropylenes and copolymers

PP:

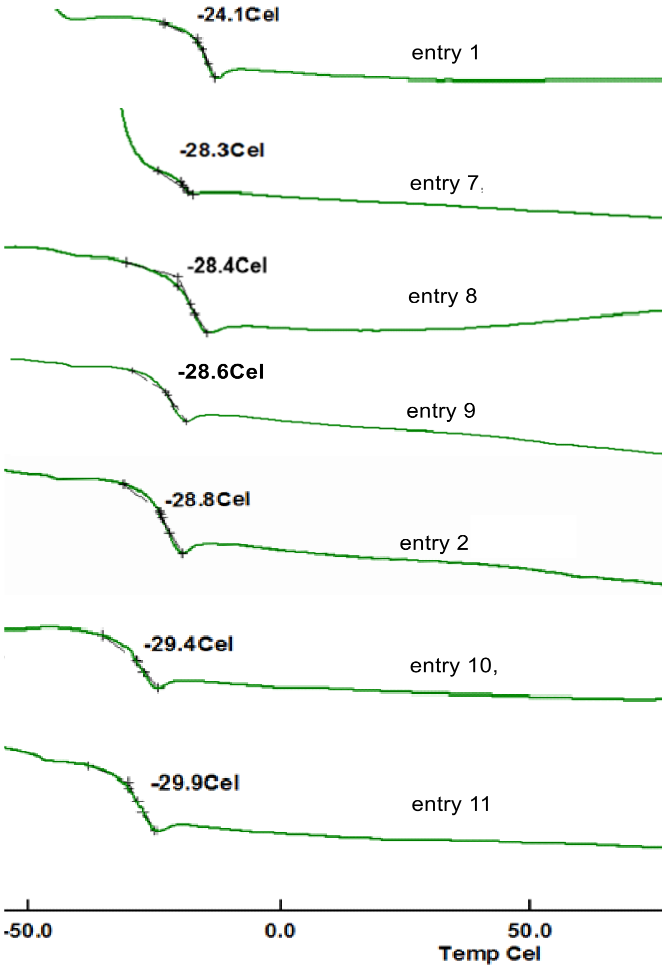

Fig. S10. DSC curves of the PPs prepared with **2** and **5** (entries 1, 2 and 7–11, Table 2).

PP-*co*-PD:

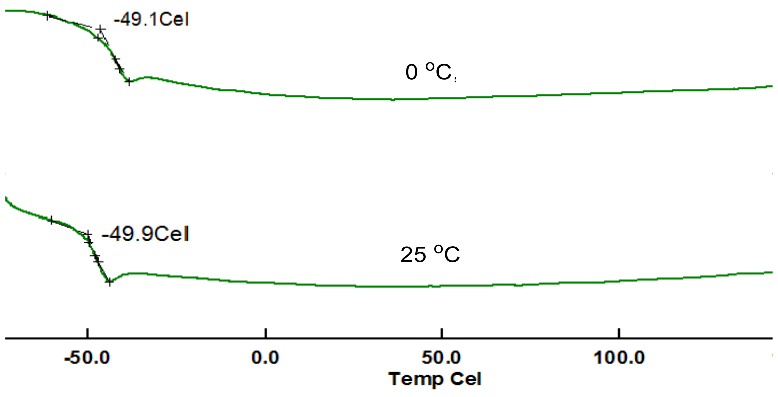

Fig. S11. DSC curves of poly(P-*co*-D)s prepared by **2**-MMAO at 0 and 25 °C (entries 1 and 2, Table 3).

9. References

S1 Wang, F. Z.; Tanaka, R.; Cai, Z. G.; Nakayama, Y.; Shiono, T. Synthesis of highly branched polyolefins using phenyl substituted α-diimine Ni (II) catalysts. *Polymers* **2016**, *8,* 160.

S2 Wang, F. Z.; Tanaka, R.; Cai, Z. G.; Nakayama, Y.; Shiono, T. Room-temperature Suzuki–Miyaura cross-coupling reaction with α-diimine Pd (II) catalysts. *Appl. Organometal. Chem.* **2015**, *29,* 771–776.

S3 Wang, F. Z.; Tanaka, R.; Li, Q. S.; Nakayama, Y.; Shiono, T. Chain-Walking Polymerization of Linear Internal Octenes Catalyzed by α-Diimine Nickel Complexes. *Organometallics* **2018**, *37,* 1358–1367.

S4 Azoulay, J. D.; Bazan, G. C.; Galland, G. B. Microstructural characterization of poly (1-hexene) obtained using a nickel α-keto-β-diimine initiator. *Macromolecules* **2010**, *43,* 2794–2800.

S5 McCord, E. F.; McLain, S. J.; Nelson, L. T. J.; Ittel, S. D.; Tempel, D.; Killian, C. M.; Johnson, L. K.; Brookhart, M. ^{13}C NMR Analysis of α-Olefin Enchainment in Poly(α-olefins) Produced with Nickel and Palladium α-Diimine Catalysts. *Macromolecules* **2007**, *40,* 410–420.
